# Genetic architecture of kernel-related traits in sweet and waxy maize revealed by genome-wide association analysis

**DOI:** 10.3389/fgene.2024.1431043

**Published:** 2024-09-27

**Authors:** Jingtao Qu, Diansi Yu, Wei Gu, Muhammad Hayder Bin Khalid, Huiyun Kuang, Dongdong Dang, Hui Wang, Boddupalli Prasanna, Xuecai Zhang, Ao Zhang, Hongjian Zheng, Yuan Guan

**Affiliations:** ^1^ CIMMYT-China Specialty Maize Research Center, Crop Breeding and Cultivation Research Institute, Shanghai Academy of Agricultural Sciences, Shanghai, China; ^2^ National Research Center of Intercropping, The Islamia University of Bahawalpur, Bahawalpur, Pakistan; ^3^ International Maize and Wheat Improvement Center (CIMMYT), Nairobi, Kenya; ^4^ International Maize and Wheat Improvement Center (CIMMYT), Texcoco, Mexico; ^5^ Shenyang City Key Laboratory of Maize Genomic Selection Breeding, College of Bioscience and Biotechnology, Shenyang Agricultural University, Shenyang, China

**Keywords:** kernel size, kernel weight, NGS, GWAS, GS

## Abstract

**Introduction:**

Maize (*Zea mays* L.) is one of the most important crops worldwide, the kernel size-related traits are the major components of maize grain yield.

**Methods:**

To dissect the genetic architecture of four kernel-related traits of 100-kernel weight, kernel length, kernel width, and kernel diameter, a genome-wide association study (GWAS) was conducted in the waxy and sweet maize panel comprising of 447 maize inbred lines re-sequenced at the 5× coverage depth. GWAS analysis was carried out with the mixed linear model using 1,684,029 high-quality SNP markers.

**Results:**

In total, 49 SNPs significantly associated with the four kernel-related traits were identified, including 46 SNPs on chromosome 3, two SNPs on chromosome 4, and one SNP on chromosome 7. Haplotype regression analysis identified 338 haplotypes that significantly affected these four kernel-related traits. Genomic selection (GS) results revealed that a set of 10,000 SNPs and a training population size of 30% are sufficient for the application of GS in waxy and sweet maize breeding for kernel weight and kernel size. Forty candidate genes associated with the four kernel-related traits were identified, including both *Zm00001d000707* and *Zm00001d044139* expressed in the kernel development tissues and stages with unknown functions.

**Discussion:**

These significant SNPs and important haplotypes provide valuable information for developing functional markers for the implementation of marker-assisted selection in breeding. The molecular mechanism of *Zm00001d000707* and *Zm00001d044139* regulating these kernel-related traits needs to be investigated further.

## 1 Introduction

Maize (*Zea mays L*.) is one of the most important cereal crops globally. In 2022, maize production reached 1,163.5 million tons, surpassing other key crops such as rice (776.5 million tons) and wheat (880.4 million tons), according to the Food and Agriculture Organization of the United Nations (https://www.fao.org/statistics/en/). Maize kernel size and weight are the two major components associated with grain yield. Specific kernel-related traits including 100-kernel weight (HKW), kernel length (KL), kernel width (KW), and kernel thickness (KT), play an important role in determining overall grain yield in maize (Gupta et al., 2006).

Genome-wide association study (GWAS) is a powerful approach to unravel the genetic basis of complex traits, which has been widely applied to various crops (Pang et al., 2020; Shikha et al., 2021; Shook et al., 2021; Soleimani et al., 2022). Maize has a high-level genetic diversity and harbors rare alleles in the genome. GWAS is an ideal tool to study the genetic architecture of complex traits in maize (Myles et al., 2009; Yan et al., 2009; Zhang X. et al., 2022). Over recent decades, numerous SNPs or candidate genes associated with these traits have been identified using this method. For example, Zhang et al. (2017) discovered 25 SNPs significantly associated with kernel weight (HKW), kernel row number (KRN), and kernel size in a study involving 240 maize inbred lines (Zhang et al., 2017). Moreover, GWAS was applied to identify 29 SNPs significantly associated with four kernel-related traits (Hao et al., 2019). Additionally, 21 SNPs and 7 SNPs were significantly associated with HKW and kernel weight efficiency (KWE), respectively (Zhang et al., 2020). Thus, exploring the genetic basis of kernel traits in maize using GWAS is crucial for enhancing crop improvement strategies.

The kernel size and weight are affected by key genes in the regulatory pathway involving cell proliferation and expansion at the kernel development stage, and several key regulatory factors involved in various signaling pathways have been identified in several previous studies. In the ubiquitin-proteasomal pathway, the *GW2* gene encoding a RING-E3 ubiquitin ligase negatively affects maize kernel size and weight (Li et al., 2010a). A recent discovery highlighted the *ZmKW1* gene, which codes for a SINA protein with E3 ubiquitin ligase activity, as a regulator of kernel weight and shape (Zhang et al., 2024). In the G-protein signaling pathway, the maize ortholog of the *GS3* gene, which encodes the γ subunit of G-protein, negatively regulates kernel size and weight and also affects the *GW2* gene (Li et al., 2010b). In the MAPK signaling pathway, the *OPAQUE11* gene, which encodes an endosperm-specific bHLH transcription factor, activates the *Zmyada* gene upstream of MAPK (Feng et al., 2018). In the phytohormone pathway, the genes *ZmYuc1/De18*, *ZmVPS29*, and *ZmSK2* have been proven to regulate kernel size and weight through the IAA signaling pathway (Bernardi et al., 2012; Chen et al., 2020; Wang et al., 2022).

Genomic Selection (GS) can help breeders improve breeding efficiency by saving phenotyping costs and reducing the breeding cycle time (Meuwissen et al., 2001; Crossa et al., 2014; Qu et al., 2022). GS utilizes a training population to estimate the effect of genetic markers based on phenotypic and genotypic data, which then helps predict the genomic estimated breeding values (GEBV) of individuals in the prediction population (Liu et al., 2023). GS has been extensively applied in maize to select desirable traits in inbred lines and to predict the performance of hybrids (Liu et al., 2017; Liu et al., 2021; Wang B. et al., 2020; Wang et al., 2020b; Song et al., 2024). The preliminary GS analysis is needed to better understand how to improve the kernel-related traits in waxy and sweet maize breeding.

In the present study, the genetic architecture of kernel-related traits in sweet and waxy maize was dissected in a GWAS including 230 waxy maize inbred lines, 112 sweet maize inbred lines, and 105 sweet-waxy maize inbred lines. The genetic loci and candidate genes regulating HKW, KL, KW, and kernel diameter (KD) were identified by GWAS, alongside a GS analysis was carried out. The present study aims to improve the understanding of the genetic architecture of kernel-related traits in waxy and sweet maize. Furthermore, it aims to contribute to the enhancement of kernel yield in both waxy and sweet maize breeding.

## 2 Materials and methods

### 2.1 Plant materials, field planting, and phenotyping

In this study, 447 maize lines, including 230 sweet maize inbred lines, 112 waxy maize lines, and 105 sweet-waxy maize lines, were used for GWAS analysis. The plants were cultivated at the Shanghai Academy of Agricultural Sciences’ experimental stations in Linshui, Hainan (110°05′E, 18°55′N) in 2020 and Zhuanghang, Fengxian District, Shanghai (121°39′E, 30°89′N) in 2021. All experimental trials followed a randomized complete block design with three replications at each location. Approximately 50 seeds from each plant were collected to evaluate the four kernel-related traits, including HKW, KL, KW, and KD using the SC-G automatic seed tester and a thousand-kernel weight scale (http://www.wseen.com/ProductDetail.aspx?id=16&classid=28). For subsequent GWAS and GS analyses, the phenotypic values of each line were represented by the best linear unbiased prediction (BLUP) values, calculated using META-R (version: 6.0, http://hdl.handle.net/11529/10201) software (Alvarado et al., 2020).

### 2.2 Sequencing and SNP calling

For genotyping, next-generation sequencing (NGS) was employed to analyze the genotypes of 447 maize inbred lines. Genomic DNA was isolated using a modified CTAB method. Each inbred line of the associated population was genotyped using Illumina sequencing technology at a 5-fold depth by Novogene Co., Ltd., Beijing, China (https://cn.novogene.com/). The raw data from Novogene were initially processed using Fastp software (version: 0.20.1, https://github.com/OpenGene/fastp) with parameters set to “-q 20 --length_required = 50” (Chen et al., 2018). The processed data were then aligned to the Maize B73 RefGen_v4 reference genome using bwa-mem software (version: 0.7.17, https://github.com/lh3/bwa) with the ‘-M’ parameter (Chen et al., 2018). The alignment results were sorted, and duplicates were marked using samtools (version: 1.6, https://www.htslib.org/) and Picard (version: 2.18.29, https://broadinstitute.github.io/picard/) software, respectively (Danecek et al., 2021). SNPs were identified using Freebayes software (version: 1.3.6, https://github.com/freebayes/freebayes) with default parameters (Garrison and Marth, 2012). The raw SNPs were filtered to retain only those with a missing rate (MR) < 5%, minor allele frequency (MAF) > 0.05, and heterozygosity rate (HR) < 0.2, using an in-house Perl script. After filtering, 1,684,029 high-quality SNPs were retained for further analysis.

### 2.3 Genome-wide association analysis and haplotype analysis

A total of 1,684,029 high-quality SNPs, evenly distributed across the ten maize chromosomes, were retained for subsequent linkage disequilibrium (LD) calculation and GWAS analysis. LD analysis was conducted using TASSEL5 software (version: 5.2.90, https://tassel.bitbucket.io/), and LD decay visualization was achieved using an R programming script provided by Zhang X. et al. (2022) (Bradbury et al., 2007; Zhang A. et al., 2022). GWAS analysis on the four kernel-related traits including HKW, KL, KW, and KD was carried out with GEMMA software (version: 0.98.5, https://github.com/genetics-statistics/GEMMA) employing a mixed linear model (MLM) (Zhou and Stephens, 2012). The number of effective SNPs, calculated using GEC software (version: 1.0, https://pmglab.top/gec/#/), determined the *p-value* threshold (Li et al., 2012). Manhattan and QQ plots were generated using CMplot (https://github.com/YinLiLin/CMplot) packages in R.

SNPs with a *p-value* < 1 × 10^−3^ from the GWAS were used for developing haplotype and subsequent haplotype-trait regression analysis. The development of haplotype was performed using LDBlockShow software (version: 1.40, https://github.com/BGI-shenzhen/LDBlockShow) with default parameters (Dong et al., 2021). The identified haplotype blocks were used to carry out the haplotype-trait regression (HTR) analysis with four kernel-related traits using stepwise regression with forward elimination in R (Rashid et al., 2022).

### 2.4 Prediction and functional annotation analysis of candidate genes

Genes located within 106.12 kb (genome-wide average distance of LD decay to *r* ^*2*^ = 0.2) around the significantly associated SNPs were selected as the candidate genes. Functional annotation of candidate genes was performed using files from MaizeGDB (https://maizegdb.org/) and agriGOV2 (http://systemsbiology.cau.edu.cn/agriGOv2/) for GO functional annotation (Tian et al., 2017). The expression level dataset was downloaded from the maizeGDB and filtered based on kernel-related tissues (Kakumanu et al., 2012; Johnston et al., 2014; Forestan et al., 2016; Stelpflug et al., 2016; Walley et al., 2016; Waters et al., 2017). The expression data of candidate genes were obtained from the filtered dataset. Finally, the expression results of candidate genes were visualized using the heatmap package in R.

### 2.5 Genomic selection analysis

The Ridge Regression Best Linear Unbiased Prediction (RRBLUP) model was employed for genomic prediction analysis (Endelman, 2011). Based on the phenotypic variation explained (PVE) values, the top 100, 500, 1,000, 3,000, 5,000, and 10,000 SNPs datasets were used to estimate the prediction accuracy for all four kernel-related traits. At each marker density, SNPs were randomly selected 500 times, and a five-fold cross-validation scheme with 500 repetitions was applied. In addition, 10%–90% of total population size, with a 10% interval, was set as the training population to explore the effect of training population size on the estimation of the prediction accuracy for all four kernel-related traits.

## 3 Results

### 3.1 Phenotypic variation and heritability of kernel size and weight

The HKW, KL, KW, and KD in the GWAS panel ranged from 9.24 to 26.80 g, 10.94–16.09 mm, 9.75–10.91 mm, and 10.32–12.32 mm, respectively, with averages of 17.44 g, 16.09 mm, 10.91 mm, and 12.32 mm (Table 1). Significant variation was observed in kernel size and weight. The broad-sense heritability (*h* ^*2*^) for these traits ranged from 0.19 to 0.87 in the GWAS panel, i.e., 0.87 for HKW, 0.54 for KL, 0.19 for KW, and 0.29 for KD. This result shows HKW and KL exhibited higher heritability, whereas KW and KD had relatively lower values. These findings indicate that HKW and KL were under stronger genetic control, whereas KW and KD were influenced to a lesser extent by genetic factors. The differences in heritability among these traits may reflect the extent of genetic variation within the population, providing valuable insights to further understand the genetic mechanisms and breeding applications of these kernel-related traits.

**TABLE 1:** The means and their standard errors (SE), genetic variances (V_G_), genotype-environment interaction variances (V_GE_), error variances (V_e_), and heritability estimates (*h* ^*2*^) for kernel-related traits across two environments in the associated panel.

Trait	Minimum	Maximum	Mean ± SE	V_G_	V_GE_	V_e_	*h* ^*2*^
HKW (g)	9.24	26.8	17.44 ± 0.19	19.1	4.42	4.04	0.87
KL (mm)	10.9	16.1	12.79 ± 0.05	2.77	4.53	0.39	0.54
KW (mm)	9.75	10.9	10.26 ± 0.01	0.38	3.12	0.31	0.19
KD (mm)	10.3	12.3	11.14 ± 0.02	0.74	3.47	0.29	0.29

**FIGURE 1:**
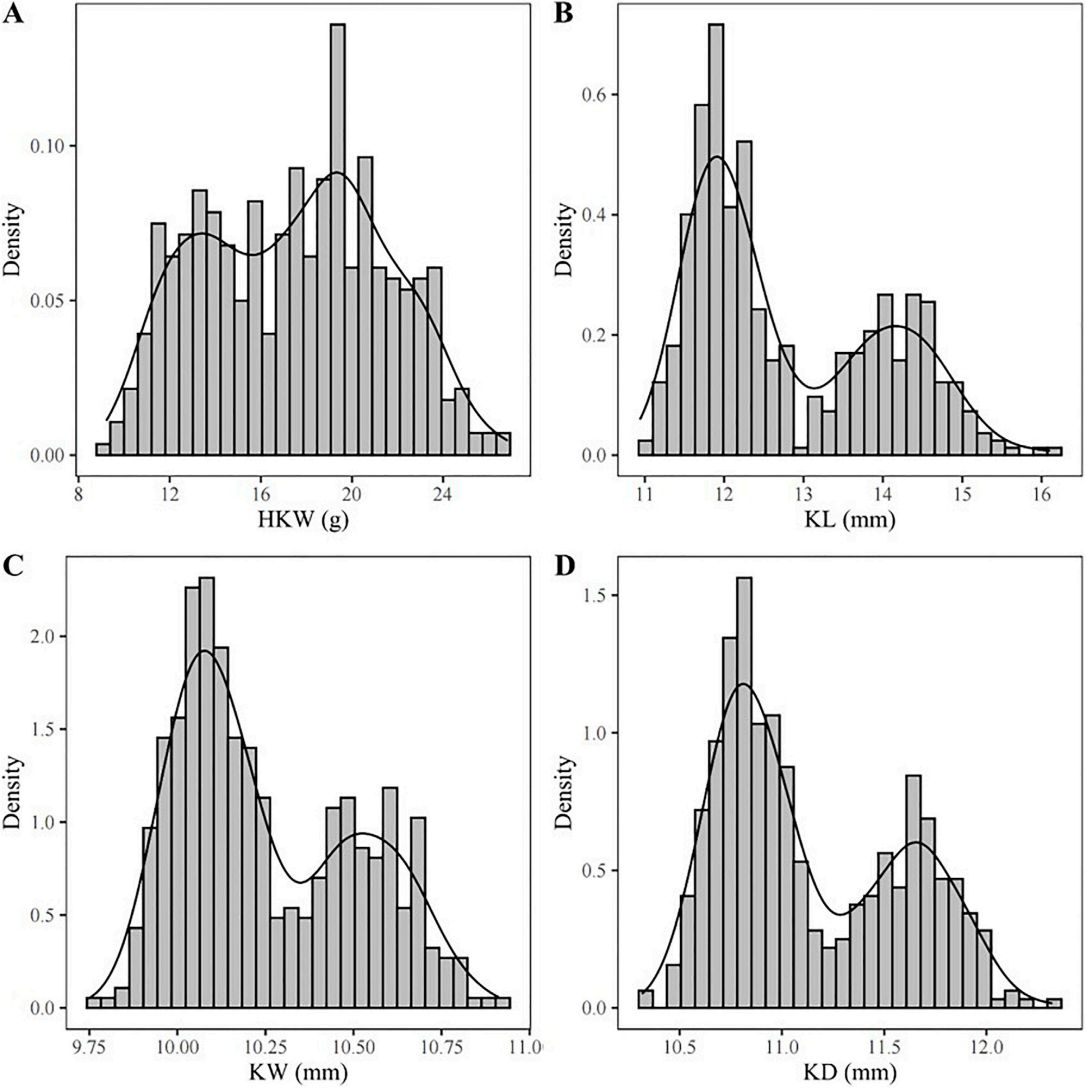
Distribution of phenotypes for HKW **(A)**, KL **(B)**, KW **(C)**, and KD **(D)** in the GWAS panel.

**FIGURE 2:**
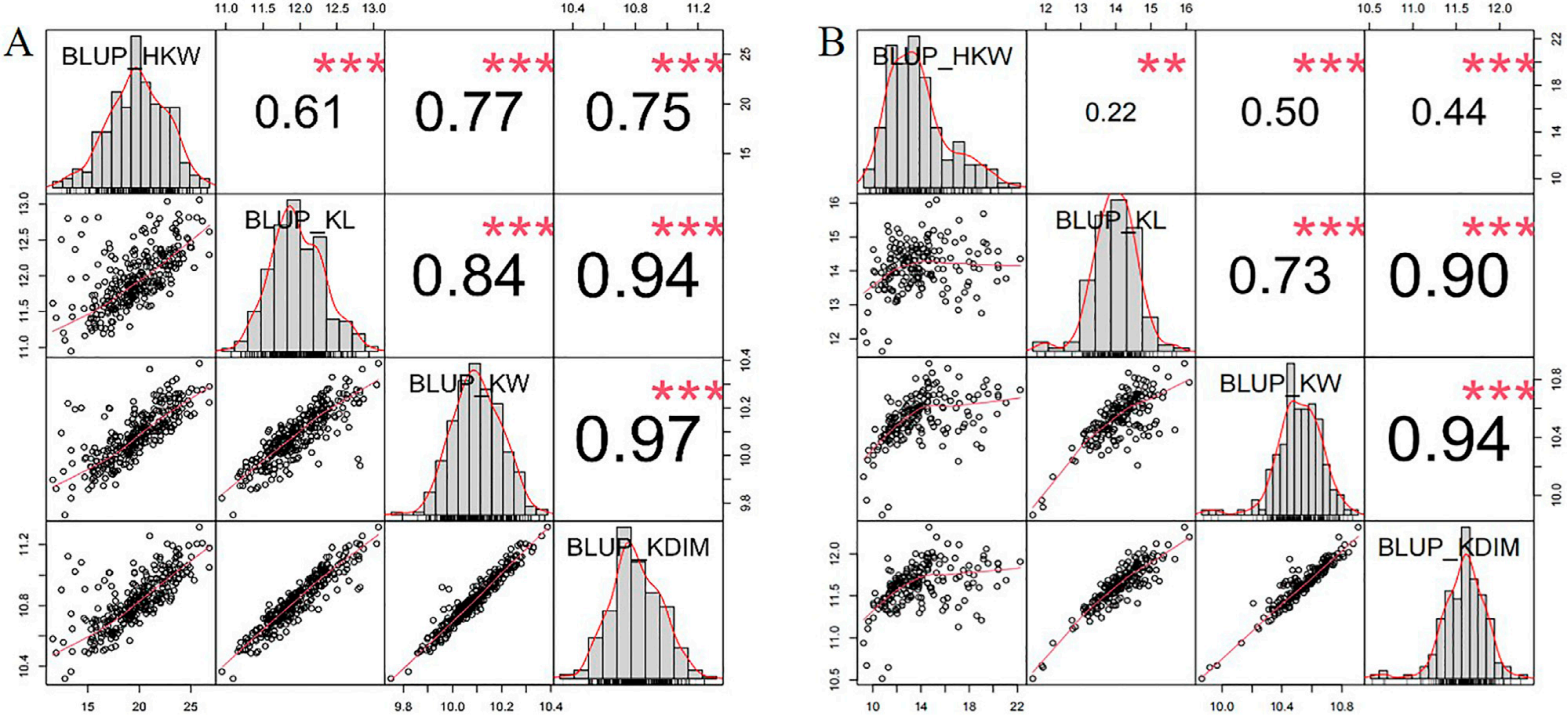
Frequency distribution and correlation analysis of HKW, KL, KW, and KD in waxy maize subgroup **(A)** and sweet maize subgroup **(B)** across two environments.

Density plots of the four kernel-related traits showed bimodal distributions (Figures 1A–D), suggesting major genes may regulate these traits in the associated panel. Additionally, the panel was divided into two subgroups based on the source information of sweet and waxy maize germplasm. Within each subgroup, correlation analyses of the traits were performed. In the waxy maize subgroup, the correlation coefficients between HKW and KL, KW, and KD were 0.61, 0.77, and 0.75, respectively (Figure 2A). In contrast, the sweet maize subgroup showed lower correlation coefficients of 0.22, 0.50, and 0.44, respectively (Figure 2B). This suggests that KL, KW, and KD were jointly contributed to regulating kernel weight in both sweet and waxy maize.

### 3.2 SNP calling, filtering, and characterizing

**FIGURE 3:**
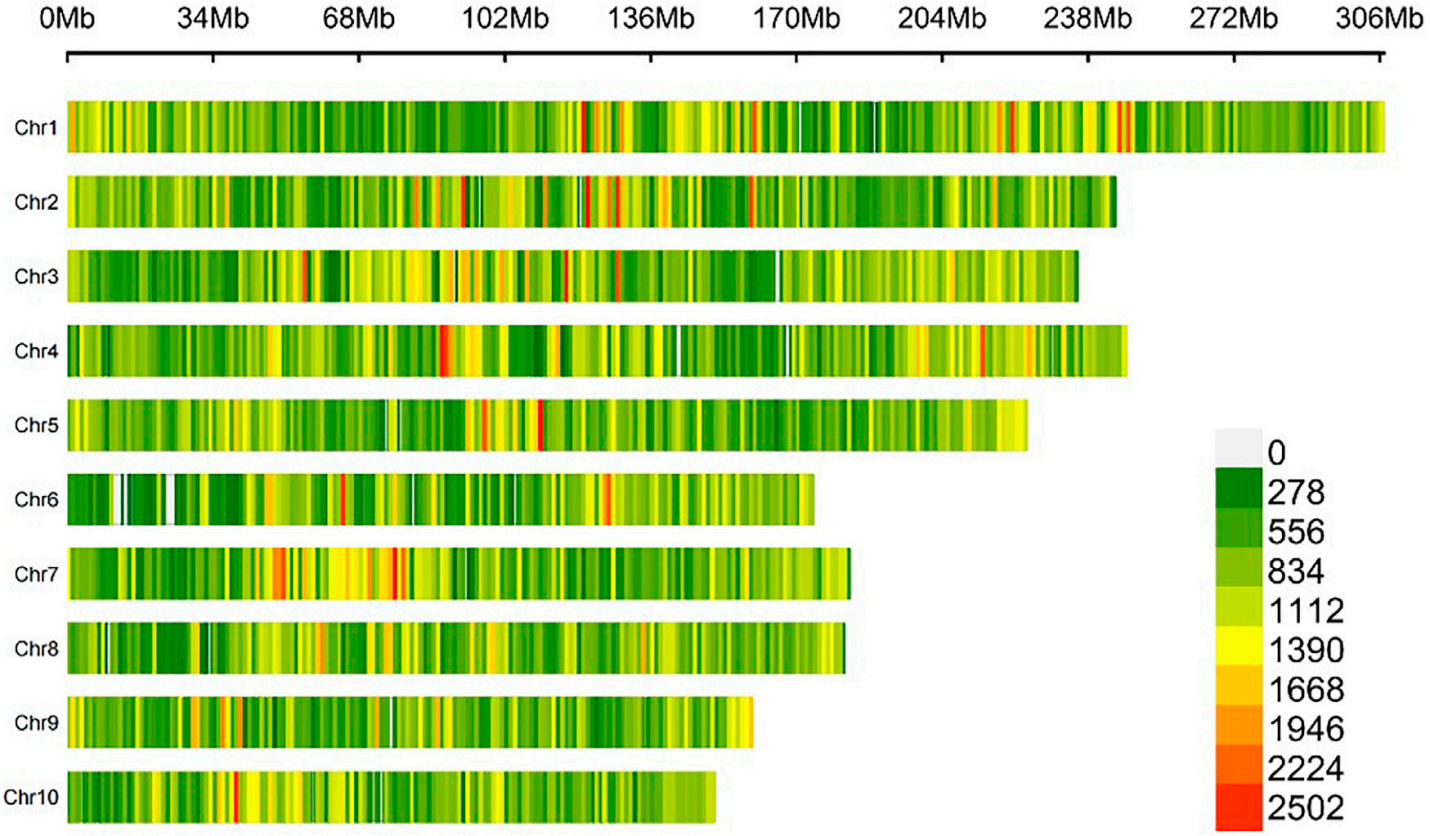
Heatmap of distribution of SNP density on ten maize chromosomes.

**FIGURE 4:**
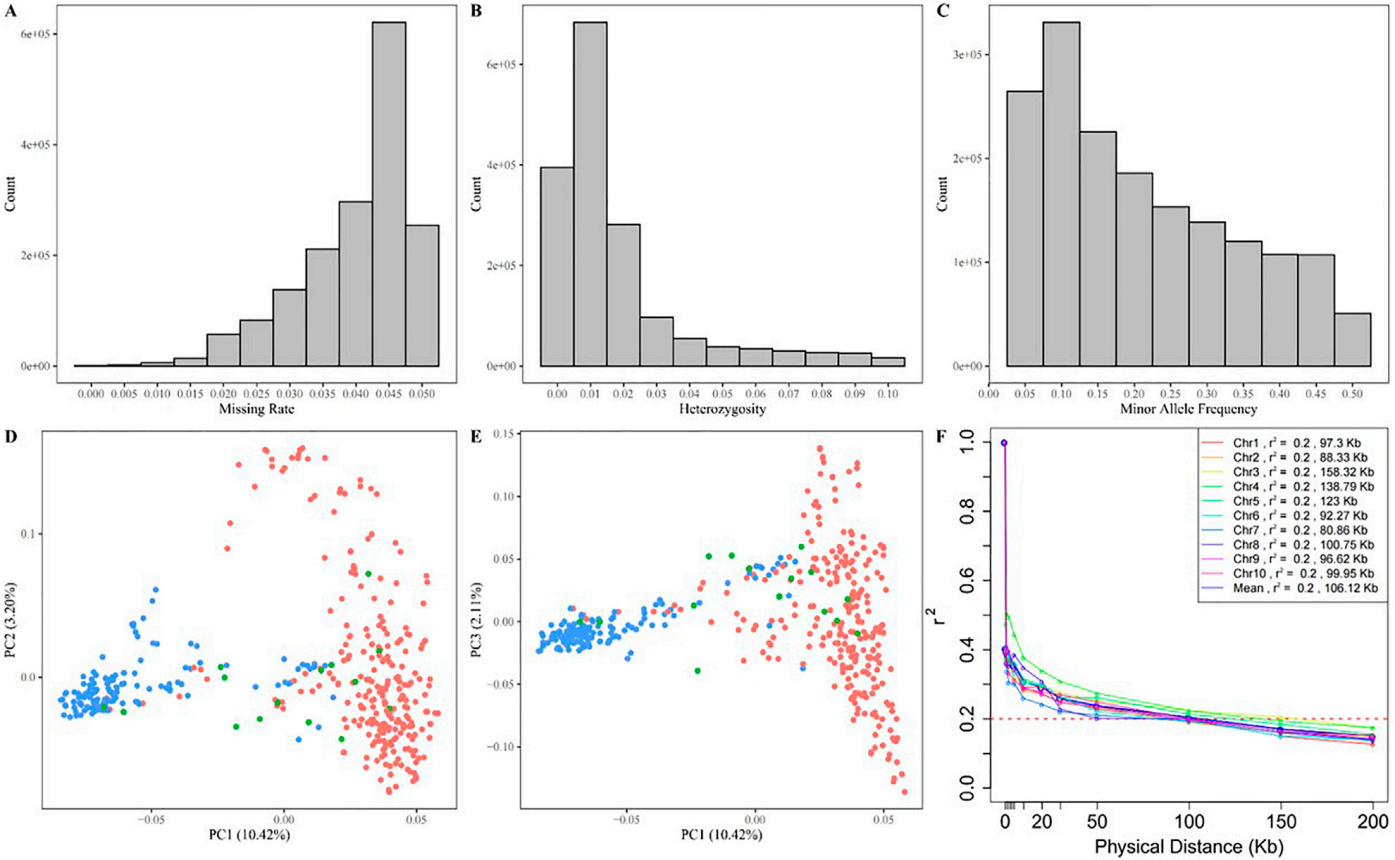
Distribution of the missing rate **(A)**, heterozygosity rate **(B)**, and minor allele frequency **(C)** of genotype. The scatter plot of the first three principal components of the GWAS panel **(D, E)**, and the LD decay plot in GWAS panel **(F)**.

The raw sequencing data were filtered, aligned to the maize B73v4 genome, and PCR duplicates were removed. This process identified 45, 728, 361 raw SNPs using Freebayes software. After filtering, 1,684,029 SNPs were retained for further analysis. The number of high-quality SNPs ranged from 120,666 on chromosome 10 to 245,799 on chromosome 1, with an average of 168,402 SNPs per chromosome. The density of these SNPs varied from 732.20 SNPs per megabase (Mb) on chromosome 6 to 834.24 SNPs/Mb on chromosome 3, averaging 799.51 SNPs/Mb, as shown in Supplementary Table S1. The high-quality SNPs were distributed relatively evenly across all ten chromosomes of the maize B73v4 genome (Figure 3). The missing rate for SNPs across 447 maize inbred lines ranged from 0 to 0.05, with an average of 0.04 (Figure 4A), while SNP heterozygosity ranged from 0 to 0.1, averaging 0.02 (Figure 4B). The minor allele frequency (MAF) ranged from 0.05 to 0.5, with an average of 0.21 (Figure 4C). This SNP dataset was deemed suitable for subsequent GWAS analysis. Within this GWAS panel, the LD decay distance was 106.12 kb at r^2^ = 0.2, estimated with this high-quality SNP dataset (Figure 4F).

Principal component analysis revealed that the first three components accounted for 15.73% of the total SNP variance (10.42% for PC1, 3.20% for PC2, and 2.11% for PC3), clearly classifying the 477 maize inbred lines into two subgroups corresponding to the original information of the sweet and waxy maize germplasm (Figures 4D, E). The sweet-waxy maize inbred lines were divided into either sweet maize subgroup or waxy maize subgroup, rather than being grouped into a separate subgroup. These findings reflect a correlation between domestication levels and selection intensity, leading to reduced genetic diversity.

### 3.3 Genome-wide association analysis and haplotype traits regression analysis

**FIGURE 5:**
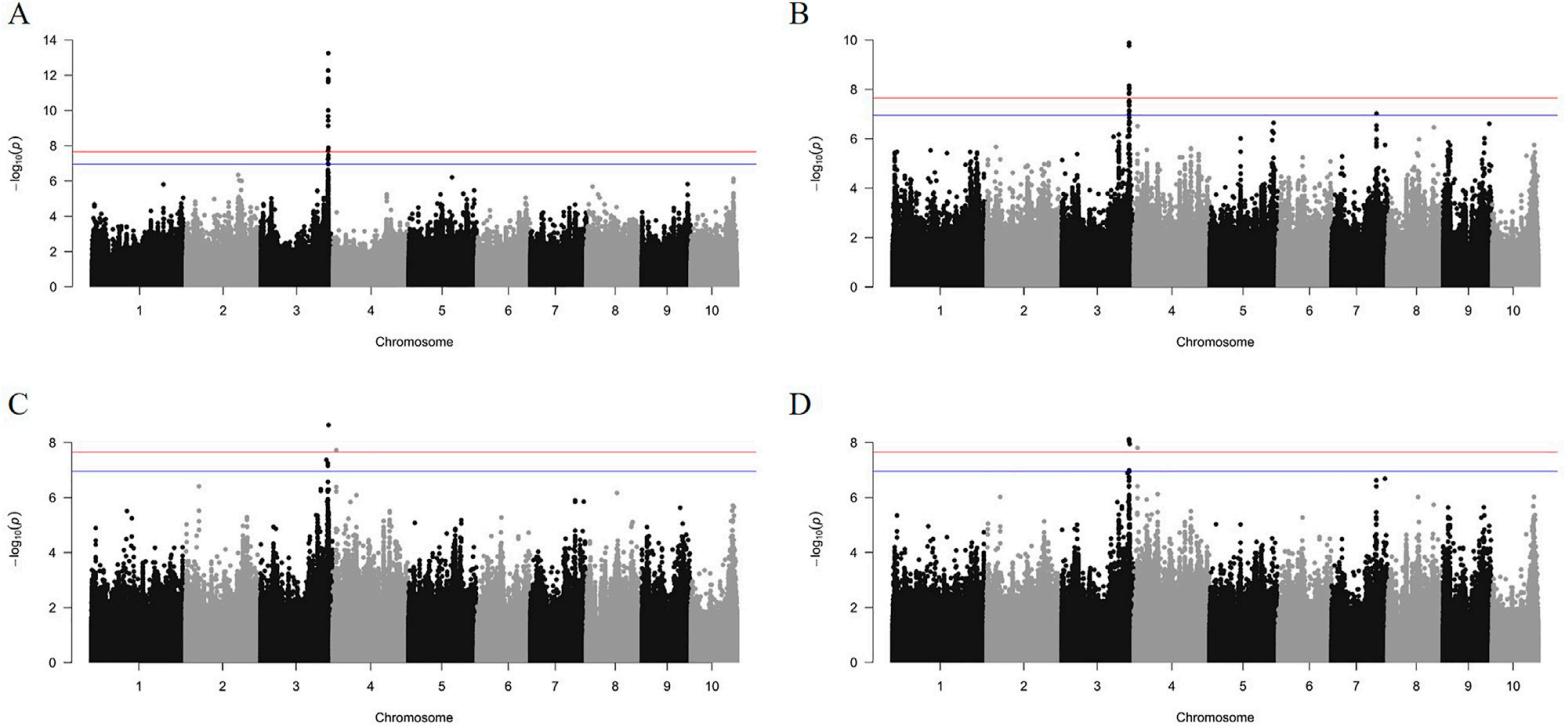
The Manhattan plots of GWAS results for HKW **(A)**, KL **(B)**, KW **(C)**, and KD **(D)** using the mixed linear model. The cut-off of 0.01/number of effective SNPs and 0.05/number of effective SNPs are represented by red and blue parallel lines, respectively.

**FIGURE 6:**
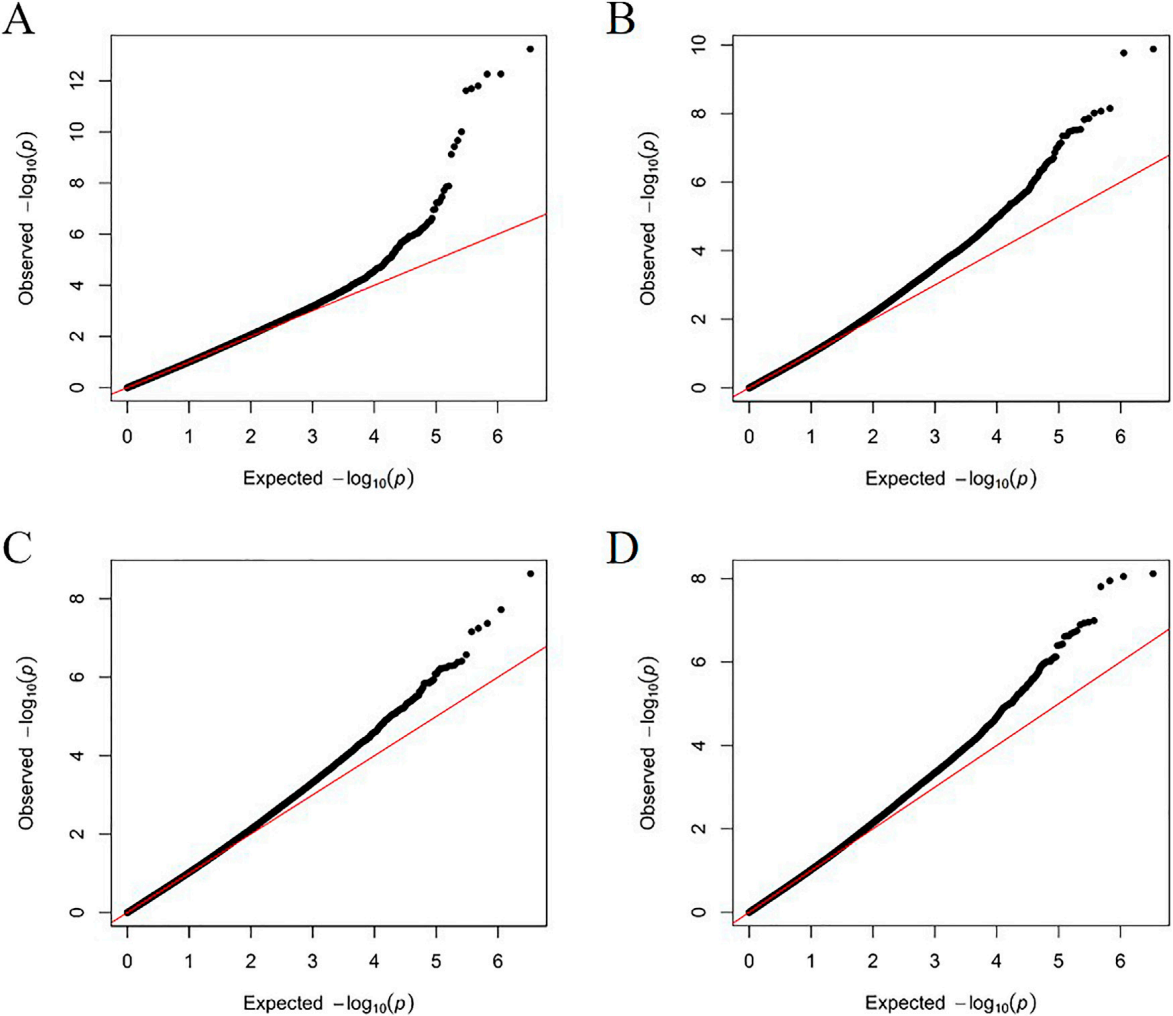
The quantile-quantile (QQ) plots of GWAS results for HKW **(A)**, KL **(B)**, KW **(C)**, and KD **(D)** using the mixed linear model.

The GWAS analysis results from the GEMMA software showed that GWAS effects for HKW, KL, KW, and KD were 17.48, 12.77, 10.25, and 11.13, respectively. Meanwhile, the total PVE for HKW, KL, KW, and KD was 73.32%, 60.33%, 50.07%, and 52.05%, respectively (Table 2). After analyzing with the GEC software, the number of effective SNP in the association panel was 445,324. The threshold of *p-value* was set to 1.12 × 10^−7^, based on the number of effective SNPs and familywise error rate of α = 0.05. A total of 49 SNPs were significantly associated with the four kernel-related traits identified by GEMMA with the MLM model (Figures 5, 6). Out of these 49 SNPs, 19 were significantly associated with HKW, 19 with KL, 5 with KW, and 6 with KD (Supplementary Table S2). These significant SNPs were located on chromosome 3, 4, and 7, with counts of 46, 2, and 1, respectively. The PVE of these significant SNPs ranged from 6.27% to 12.33%, with an average value of 7.73%. The maximum PVE values were 12.33% (S3_220335807) for HKW, 9.25% (S3_220227161) for KL, 7.95% (S3_222360985) for KW, and 7.55% (S3_220227161) for KD. Meanwhile, the minimum *p-values* for HKW, KL, KW, and KD were 5.66 × 10^−14^, 1.30 × 10^−10^, 2.30 × 10^−9^, and 7.62 × 10^−9^, respectively.

**TABLE 2:** The PVE and their standard errors (SE), genetic variances (V_G_), error variances (V_*e*_), and Beta and their standard errors (SE) for GWAS using mix liner model in the associated panel.

Traits	PVE ± SE (%)	V_G_	V_e_	Beta ± SE
HKW	73.32 ± 5.58	7.83	2.85	17.48 ± 0.08
KL	60.33 ± 5.18	0.34	0.22	12.77 ± 0.02
KW	50.07 ± 5.68	0.02	0.02	10.25 ± 0.01
KD	52.05 ± 5.52	0.05	0.04	11.13 ± 0.01

After applying a filtering criterion of *p-value* < 0.001, the numbers of retained SNPs were 1,808 for HKW, 3,213 for KL, 2,350 for KW, and 2,550 for KD. These were used in haplotype analyses conducted using the LDblockshow software. The analysis formed 316, 438, 338, and 370 haplotype blocks for HKW, KL, KW, and KD, respectively, which were then used for HTR analyses using BLUP for the kernel-related traits (Supplementary Table S4). The HTR analysis detected 61, 122, 66, and 89 significant haplotypes for HKW, KL, KW, and KD, respectively, with adjusted *p-values* < 0.05. The PVE values of the haplotypes ranged from 1.10% to 6.28% for HKW, 1.09%–11.76% for KL, 1.21%–6.78% for KW, and 1.17%–10.19% for KD (Supplementary Table S5). Notably, haplotype of hap6.16 for HKW on chromosome 6, comprising two SNPs at 142 Mb (S6_142324685 and S6_142324939), had the lowest adjusted *p-value* of 1.01 × 10^−5^. Similarly, haplotype of hap4.62 for KL on chromosome 4, formed by 51 SNPs at 239 Mb, had the lowest adjusted *p-value* of 4.19 × 10^−11^. Haplotype of hap3.10 for KW on chromosome 3, consisting of two SNPs at 161 Mb, and haplotype of hap9.23 for KD on chromosome 9, formed by six SNPs at 129 Mb, also demonstrated significantly low adjusted *p-values* of 4.19 × 10^−11^ and 2.20 × 10^−9^, respectively.

### 3.4 Functional annotation of candidate genes

**FIGURE 7:**
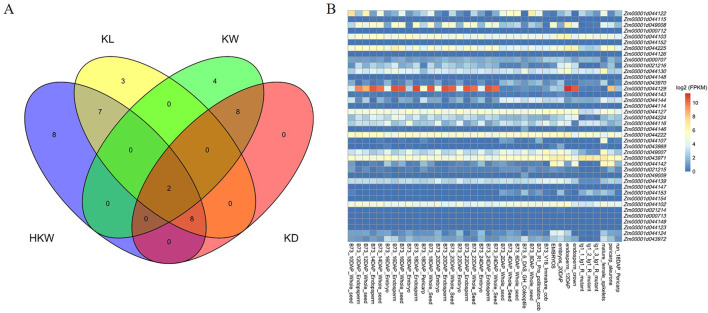
**(A)** Venn plot of candidate genes overlapping among the four kernel-related traits. **(B)** The expression heatmap of the candidate genes in kernel-related tissues.

In total, 40 candidate genes were identified in the genomic regions spanning 106.12 kb upstream and downstream of the significant associated SNPs, and the annotation of candidate genes was performed using the B73 RefGen_v4 as the reference genome. Among them, the number of candidate genes identified for HKW, KW, KL, and KD was 25, 20, 14, and 18, respectively (Figure 7A). A dataset comprising expression levels from 194 maize tissues was downloaded from MaizeGDB and refined to include data from 34 kernel-related tissues. From this refined dataset, expression data for 40 candidate genes were extracted (Figure 7B). The gene *Zm00001d044129* exhibited the highest expression with an FPKM value of 1,599.63 in the endosperm tissue 16 days after pollination in the B73 inbred line. Among the 40 candidate genes, only *Zm00001d000713*, *Zm00001d044149*, and *Zm00001d044123* did not exhibit expression in these kernel-related tissues. In contrast, the remaining 37 genes were expressed in these tissues (FPKM > 1).

Function annotations were available for 26 of the 40 candidate genes (Supplementary Table S3). *Zm00001d000707* and *Zm00001d044139* were associated with all four kernel-related traits. Notably, *Zm00001d044129* and *Zm00001d044143*, encoding glucose-1-phosphate adenylyltransferase and ubiquitin carboxyl-terminal hydrolase 27 respectively, have been reported for regulation of HKW and KL. Mutations in *Zm00001d044129* caused maize kernel shrinkage. These genes can directly or indirectly regulate the weight and size of maize kernels.

Thirty-five candidate genes were annotated with 174 GO terms: 105 for biological processes, 35 for cellular components, and 34 for molecular functions (Supplementary Table S6). These terms include signal transduction (GO:0007165), multicellular organism development (GO:0007275), regulation of hormone levels (GO:0010817), signaling (GO:0023052), developmental processes (GO:0032502), hormone metabolic process (GO:0042445), and cellular developmental process (GO:0048869), which may be involved in maize kernel development.

### 3.5 Estimation of genomic prediction accuracies

**FIGURE 8:**
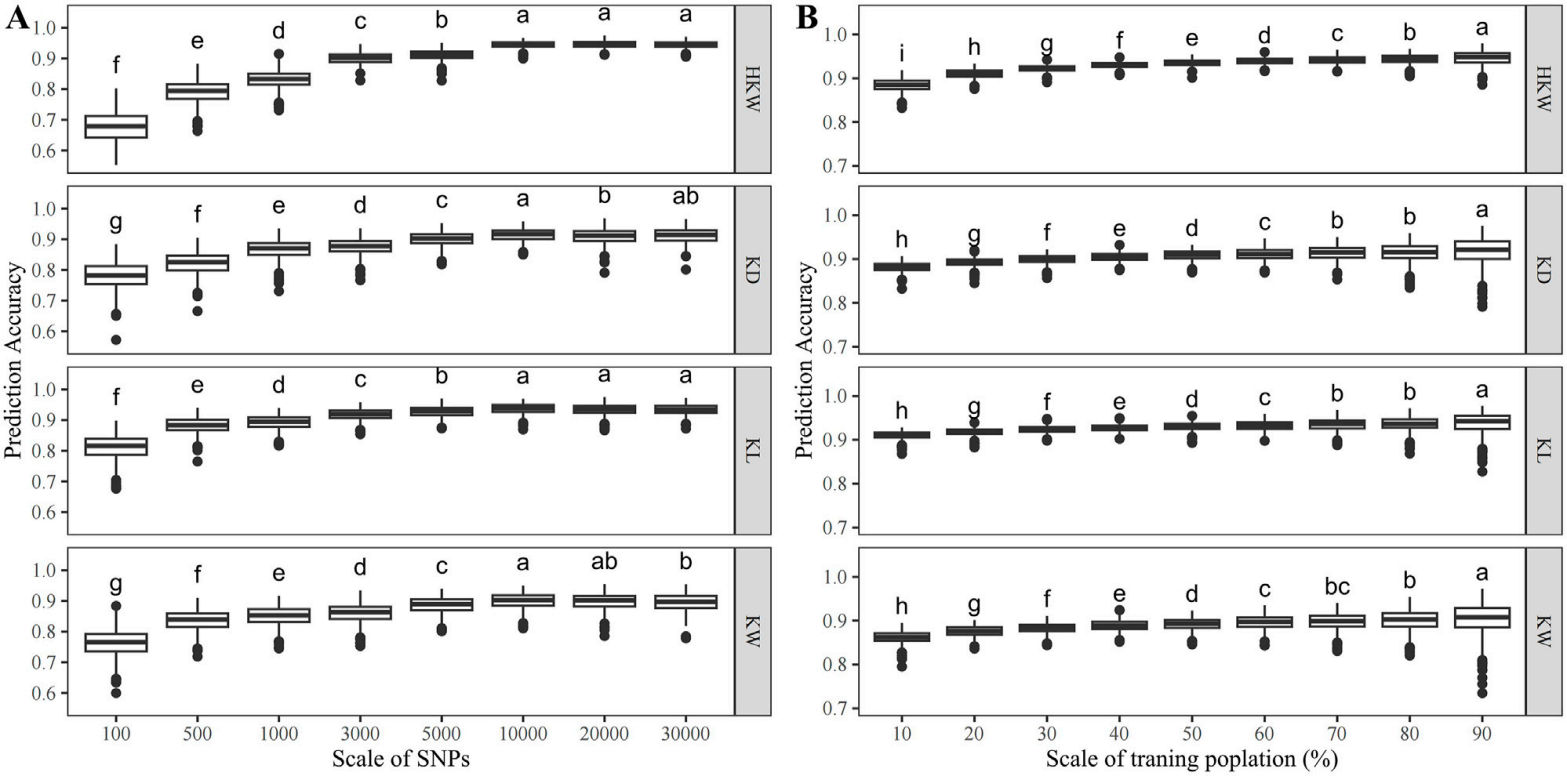
Genomic prediction accuracy of HKW, KL, KW, and KD in the population **(A)** when the number of SNPs varied from 100 to 30,000 with 8 scales, **(B)** when the training population size ranged from 10% to 90% of the total population size.

The accuracy of genomic prediction improves as the number of SNPs increases (Figure 8A). For HKW, the prediction accuracy increased from 0.67 with 100 SNPs to 0.94 with 10,000 SNPs. For KL, it rose from 0.81 with 100 SNPs to 0.94 with 10,000 SNPs. For KW, the prediction accuracy increased from 0.76 with 100 SNPs to 0.90 with 10,000 SNPs. For KD, it went from 0.78 with 100 SNPs to 0.91 with 10,000 SNPs (Table 3). At a scale of 10,000 SNPs, the prediction accuracy for all traits is consistently above 0.90.

**TABLE 3:** Prediction accuracy for four maize kernel-related traits across different scale of SNP.

Scale of SNP	Prediction accuracy			
	HKW	KL	KW	KD
100	0.67	0.81	0.76	0.78
500	0.79	0.88	0.84	0.82
1,000	0.83	0.89	0.85	0.87
3,000	0.90	0.92	0.86	0.88
5,000	0.91	0.93	0.89	0.90
10,000	0.94	0.94	0.90	0.91

The accuracy of genomic prediction also improves with the increase of the training population size (Figure 8B). For HKW, the prediction accuracy increased from 0.88 with 10% of the GWAS panel as the training population to 0.95 with 90%. For KL, the prediction accuracy increased from 0.91 with 10% of the GWAS panel as the training population to 0.94 with 90% of the GWAS panel as the training population. For KW, it rose from 0.86 with 10% of the GWAS panel as the training population to 0.90 with 90% of the GWAS panel as the training population. For KD, the prediction accuracy increased from 0.88 with 10% of the GWAS panel as the training population to 0.92 with 90% of the GWAS panel as the training population (Table 4). The prediction accuracy for the four traits tends to saturate, when more than 60% of the GWAS panel was used as the training population.

**TABLE 4:** Prediction accuracy for four maize kernel-related traits across different scale of training population.

Scale of training population (%)	Prediction accuracy			
	HKW	KL	KW	KD
10	0.88	0.91	0.86	0.88
20	0.91	0.92	0.88	0.89
30	0.92	0.92	0.88	0.90
40	0.93	0.93	0.89	0.90
50	0.93	0.93	0.89	0.91
60	0.94	0.93	0.90	0.91
70	0.94	0.93	0.90	0.91
80	0.94	0.94	0.90	0.91
90	0.95	0.94	0.90	0.92

These results revealed that a set of 10,000 SNPs and a training population size of 30% are sufficient for the application of GS in waxy and sweet maize breeding for kernel weight and kernel size.

## 4 Discussion

The kernel weight and size are among the most crucial factors influencing grain yield in maize (Liu et al., 2017). In the present study, GWAS, HTR, and GS analyses were conducted to dissect the genetic architecture of four kernel-related traits of HKW, KL, KW and KD in a representative sweet and waxy maize inbred line panel. The phenotypic analysis results of the four kernel-related traits across two environments revealed that the heritability of HKW and KL is moderately high, being 0.87 and 0.54, respectively. Both the genetic effects and genetic-environment interaction are significant, while the environmental effects are not significant, indicating that these two traits are primarily influenced by genetic factors. On the other hand, KW and KD exhibit lower heritability, with values of 0.19 and 0.29, respectively. This may be attributed to the relatively small phenotypic variation of these traits in this GWAS panel. The observed differences in heritability among these traits may reflect varying levels of genetic variation within the GWAS panel. Such insights are valuable for further understanding the genetic mechanisms underlying these kernel-related traits and can inform breeding strategies aimed at improving specific characteristics, particularly those with higher heritability like HKW and KL.

In GWAS, high-density and high-quality SNPs across the entire maize genome are essential to identify the SNPs significantly associated with the target traits. Genotyping by target sequencing (GBTS), genotyping-by-sequencing (GBS), and chip-based genotyping have been extensively utilized in the GWAS studies in maize (Xu et al., 2017; Wang et al., 2020c; Guo et al., 2021; Vinayan et al., 2021). Compared with these genotyping methods, whole-genome resequencing has a higher genome coverage and SNP density. With the dramatic decrease in the cost of whole-genome resequencing, this genotyping technique is being widely applied in GWAS analyses in maize (Xiao et al., 2017). In the present study, with the availability of the resequencing dataset of 447 sweet and waxy maize inbred lines, a saturated genome-wide dataset including 1,684,029 SNPs was used for GWAS analysis, the results showed the high-quality and high-density SNP dataset extracted from the re-sequencing data is powerful for obtaining more accurate results.

In this GWAS panel, PCA analysis distinctly divided the 447 maize inbred lines into two subgroups (Figures 4D,E). The results indicated that there was a very small partial germplasm exchange between sweet and waxy maize inbred lines in breeding selection. The main emphasis is on the improvement within each subgroup. Meanwhile, the LD decay distance at r^2^ = 0.2 was 106.12 kb, which is much greater than the LD decay distance of 33 kb in 350 modern maize lines developed from 2000 to 2010 in China (Wang B. et al., 2020), indicating lower genetic diversity of the GWAS panel used in the present study. Sweet and waxy maize are primarily cultivated for human consumption. The breeding process has historically emphasized traits linked to eating quality and nutritional value, resulting in decreased genetic diversity and increased linkage disequilibrium among genetic loci.

The GWAS has been shown to be an effective strategy for mining genetic loci for kernel weight and size (Liu et al., 2020). Due to the relatedness among maize inbred lines, the GWAS was conducted using the mixed linear model with the incorporation of the kinship matrices. The *p-value* of SNPs was the parameter to assess the association level between SNP and the trait. The smaller the *p-value*, the more significant the association between SNPs and the trait. To control the familywise error rate, 0.05/number of effective SNPs were used as the cut-off to ensure statistical significance for these SNPs (Li et al., 2022). In total, 49 SNPs were significantly associated with the four kernel-related traits based on the strict cut-off of the *p-value*. Candidate gene analysis revealed that 40 genes are the putative candidate genes for the four kernel-related traits. Out of these 40 genes, 37 genes were expressed in the kernel-related tissues of maize. *Zm00001d044129* encodes the ADP-glucose pyrophosphorylase that affects starch metabolism in the maize endosperm (Bhave et al., 1990). Maize with mutations in the *Zm00001d044129* gene exhibits a kernel shrunkage, affecting the weight and size of the maize kernel. Although the genes *Zm00001d000707* and *Zm00001d044139* lack functional annotations, they were found to be associated with all four kernel-related traits. Further experiments are needed to validate their functional characterization. Based on the GWAS results, functional markers can be developed, which will facilitate the marker-assisted selection to improve these kernel-related traits in sweet and waxy maize.

Genomic prediction has been successfully applied to several crops to accelerate the grain yield in maize breeding programs (Wang et al., 2020b). In the present study, the top 10,000 SNPs with the highest PVE values for each kernel-related trait were used for the GP analysis. In this GWAS panel, the predictive accuracy increased as the increase of the training population size. At a training population size of 10%, the lowest predictive accuracy for the four kernel-related traits, notably KW, reached 0.86. As the training population size increased, the prediction accuracy for HKW and KL stabilized around 0.94, while for KW and KD, it plateaued at approximately 0.91. Compared to previous approaches using SNPs significantly associated with the trait or random SNPs for GP analysis, this method exhibited higher predictive accuracy in GP analysis (Dang et al., 2023; Liu et al., 2023). Meanwhile, the predictive accuracy increased with the expansion of the SNPs from 100 to 10,000. At 500 SNPs, the predictive accuracy for the four kernel-related traits reached approximately 0.80. In this study, more than one and a half millions SNPs were used for GWAS analyses. However, our GS results showed that the prediction accuracies for all the kernel-related traits reached plateaus above 0.90, when 10,000 SNPs were used in prediction, these prediction accuracies are relatively high, indicating that using all the more than one million SNPs for prediction is not necessary. In practical applications, the KASP genotyping platform can be used (Semagn et al., 2014; Qu et al., 2022). This genotyping platform offers a faster and more cost-effective approach for low-density genotyping of a large number of individuals (500 SNPs). The prediction accuracies for four kernel-related traits were above 0.90 using 10,000 SNPs. In this case, the GBTS platform, suitable for medium-density genotyping, can be used (Guo et al., 2019).

## Data Availability

The datasets presented in this study can be found in online repositories. This datasets have been deposited in the Genome Variation Map in National Genomics Data Center, Beijing Institute of Genomics, Chinese Academy of Sciences and China National Center for Bioinformation, under accession number GVM000854 (https://ngdc.cncb.ac.cn/gvm/getProjectDetail?project=GVM000854).
